# Systemic lupus erythematosus associated with erythema multiforme: A rare case report of Rowell's syndrome

**DOI:** 10.1002/ccr3.8677

**Published:** 2024-03-26

**Authors:** Madhur Bhattarai, Niraj Kumar Sharma, Shreeram Paudel, Sujata Bhandari, Amrit Bhusal, Kiran Dhonju, Sandip Kuikel, Shivendra Kumar Jha, Egesh Aryal, Deepak Subedi

**Affiliations:** ^1^ Maharajgunj Medical Campus, Institute of Medicine Tribhuvan University Maharajgunj Nepal; ^2^ Sukraraj Tropical and Infectious Disease Hospital Kathmandu Nepal; ^3^ Nobel Medical College Teaching Hospital Biratnagar Nepal; ^4^ BP Koirala Institute of Health Sciences Dharan Nepal; ^5^ Nepalese Army Institute of Health Sciences Kathmandu Nepal

**Keywords:** antinuclear antibodies, erythema multiforme, rheumatoid factor, rowell syndrome, systemic lupus erythematosus

## Abstract

**Key Clinical Message:**

Although it is very uncommon, SLE may initially present with recurrent episodes of EM‐like rash. Despite the various possibilities underlying their association, prompt identification, and treatment of SLE in patients presenting with EM is important to prevent death or serious organ damage.

**Abstract:**

Rowell's syndrome (RS) is an uncommon presentation of systemic lupus erythematosus (SLE) with erythema multiforme (EM)‐like lesions associated with specific serological changes, including positive rheumatoid factor (RF), speckled antinuclear antibody (ANA), positive rheumatoid factor, or anti‐La antibodies in the serum. Our case, a 41‐year‐old male, presented with features of EM. Upon investigation, we identified underlying systemic lupus erythematosus, marking a rare instance of SLE presenting for the first time as EM. Classical or true EM is precipitated by trigger factors such as infective agents like the herpes simplex virus, Mycoplasma pneumoniae, drugs like anticonvulsants, antibiotics, and non‐steroid anti‐inflammatory drugs, any underlying malignancy, or connective tissue disorders, and is not associated with any specific serological abnormalities. EM cases associated with LE lesions where an EM trigger factor is missing are considered an RS diagnostic criterion. In this case report, the importance of considering SLE in patients presenting initially with recurrent episodes of EM‐like rash is emphasized. RS should be considered, especially when there is no evidence of triggering factors. Early diagnosis and prompt treatment of SLE are crucial to preventing death and irreversible organ damage.

## 1 INTRODUCTION

Rowell's syndrome (RS) is an uncommon entity in which patients with systemic lupus erythematosus (SLE) rarely develop characteristic lesions similar to those of erythema multiforme (EM)‐like skin lesions, in the presence of specific serological abnormalities.^1^, ^2^, ^3^, ^4^ Firstly, the association between lupus and EM was described by Scholtz in 1922. In 1963, Professor Neville Rowell and his colleagues reported four female patients with discoid lupus erythematosus (DLE) and EM‐like skin lesions (among 120 patients with DLE).^2^, ^3^ SLE is a chronic autoimmune‐mediated inflammatory disorder with multisystem and multiorgan manifestations, while EM is an acute, immune‐mediated condition linked to infection, medications, and autoimmune disorders without special autoantibodies, as seen in autoimmune disorders. EM is distinguished by evident target lesions on the skin, accompanied by erosion, blisters, or bullae of mucosal areas (such as the mouth, genitals, and eyes).^3^, ^5^

To reach a diagnosis, meeting all major criteria along with one minor criterion is necessary. Major criteria include the presence of systemic or cutaneous lupus erythematosus (CLE), erythema multiform‐like lesions, and antinuclear antibodies (ANA) with speckled pattern. Minor criteria include the presence of chilblains, anti‐Ro, or anti‐La antibodies or rheumatoid factor (RF).^4^ The appearance of the speckled pattern of ANA is the most reliable and consistent factor in the diagnosis of RS.^1^, ^6^ While the precise etiopathogenesis of RS remains unclear, it is believed to be triggered by factors such as drugs, infections, ultraviolet exposure, cigarette smoking, and psychological stress.^1^, ^3^ Eventually, RS is considered a rare but distinct entity in rheumatology, and systemic lupus erythematosus (SLE) presenting initially as EM‐like lesions is quite uncommon.^2^

## 2 CASE PRESENTATION

### 2.1 Case history/Examination

A 41‐year‐old male presents to the clinic with a chief complaint of fever and rashes for the past 2 days. The fever had a gradual onset and was relieved upon taking medication. The patient reports a maximum temperature of 100 °F and denies experiencing chills or rigor. The rashes initially appeared on the neck and gradually spread throughout the entire body, including the hands, legs, and feet. The rashes started as small erythematous papules and enlarged with central necrosis. The patient also reports a history of taking diclofenac tablets for 2 days, which was approximately 4 days before the onset of the rash. No other significant medical history is reported, including the absence of respiratory symptoms, chest pain, gastrointestinal symptoms, jaundice, photosensitivity, urinary symptoms, a history of rash in the past, or any history of red or frothy urine. He had no other comorbidities, such as high blood pressure, coronary heart disease, diabetes, or any other chronic illnesses. The patient denies any recent travel, intravenous drug use, or promiscuous sexual habits. The patient provides a history of using steroids after the onset of symptoms.

On physical examination, multiple violaceous plaques with central necrosis and a peripheral erythematous to hypopigmented halo are observed over the anterior neck, abdomen, posterior back, bilateral dorsum of the hands, soles, bilateral lower limbs, and feet. The patient's nails demonstrate splinter hemorrhages, nail fold erythema, and red lunula. The patient's vital signs are as follows: pulse rate of 100 beats per minute, temperature of 100 °F, blood pressure of 180/80 mmHg, and oxygen saturation level of 96%.

## 3 METHODS

Laboratory investigations reveal a hemoglobin level of 10.21 g/dL, a total leukocyte count of 4830 cells/mm^3^, and a random blood sugar level of 158 mg/dL. The erythrocyte sedimentation rate was 35 mm/hr (reference range: 0–20). The liver function test and renal function test were normal. Serological tests revealed positive antinuclear antibodies (ANA) with a speckled pattern, positive anti‐double‐stranded DNA (anti‐dsDNA) antibodies, positive rheumatoid factor (RF), negative anti‐Ro antibody, and negative anti‐La antibody. However, the tests for scrub typhus, brucella antibodies, leptospirosis, and typhoid were negative. HIV, HBsAG, and HCV were non‐reactive. The summary and detail of the entire laboratory investigations including the serological tests can be shown in Table 1.

**TABLE 1:** Table showing all the laboratory investigations performed and their results.

S.N	Investigations	Result	Normal range
**1.**	**Hemoglobin**	**10.21 g/dL**	**12–16 g/dL**
2.	White Blood Cells (WBCs)	4830 cells/mm^3^	4500–11,000 cells/mm^3^
3.	Neutrophils	50%	40%–60%
4.	Lymphocytes	27%	20R–40%
5.	Platelets	194,000/μL	150,000–450,000/μL
6.	INR	0.9 s	0.8–1.1 s
**7.**	**ESR**	**35 mm/h**	**0–20 mm/h**
**8.**	**CRP**	**6 mg/dL**	**0.3–1.0 mg/dL**
9.	Total bilirubin	0.9 mg/dL	0.1–1.2 mg/dL
10.	Direct bilirubin	0.3 mg/dL	0.0–0.3 mg/dL
11.	SGOT/AST	25 units/L	10–36 units/L
12.	SGPT/ALT	25 units/L	4–36 units/L
13.	TSH	1.20 microIU/ml	0.27–4.20 microIU/ml
14.	Creatinine	0.8 mg/dL	0.6–1.2 mg/dL
15.	BUN	16 mg/dL	6–24 mg/dL
16.	Sodium	142 mEq/L	135–145 mEq/L
17.	Potassium	4.0 mEq/L	3.5–5.2 mEq/L
**18.**	**RBS**	**158 mg/dL**	**70–140 mg/dL**
19.	HbA1c	5.0%	4.0–5.6%
20.	Triglyceride	120 mg/dL	<150 mg/dL
21.	Serology		

Serological test	Result
ANA	Positive with speckled pattern
Anti‐Ro antibody	Negative
Anti‐La antibody	Negative
RF	Positive
Anti‐ds DNA antibody	Positive
HIV	Non‐reactive
HbsAG	Non‐reactive
HCV	Non‐reactive
Scrub IgM antibody	Negative
Brucella IgG and IgM antibodies	Negative
Leptospira IgM antibody	Negative
IgG and IgM for typhoid	Negative

*Note*: Abnormal values in the corresponding parameters are bolded.

A wound swab was sent for culture, which came sterile. A skin biopsy was performed on the right forearm, and the histopathological examination revealed a skin fragment lined by keratinized stratified squamous epithelium. The biopsy showed areas of ulceration, follicular plugging, and mixed inflammatory cell exocytosis. As shown in Figure 4, the dermis exhibited edema and hemorrhage, with thrombi formation and degenerated endothelial cells in a few vessels. Dense mixed inflammatory infiltrates were observed throughout the dermis, primarily in the peripilar unit, including the perivascular region. The erector pili muscle appeared unremarkable, while the subcutis was scant and unremarkable. Focal areas showed subepidermal bulla and lymphocytic infiltration at the dermo‐epidermal junction.

Based on the patient's clinical presentation, including the fever, a characteristic rash with central necrosis and peripheral halo, oral ulcers, nail hemorrhages, positive anti‐dsDNA antibodies, and a positive ANA with a speckled pattern, the initial differential diagnoses to consider include systemic lupus erythematosus (SLE) and bullous erythema multiforme.

Our patient was subsequently managed with hydroxychloroquine, prednisolone, steroid ointment, a proton pump inhibitor, and sunscreen cream after the diagnosis.

## 4 RESULTS

**FIGURE 1:**
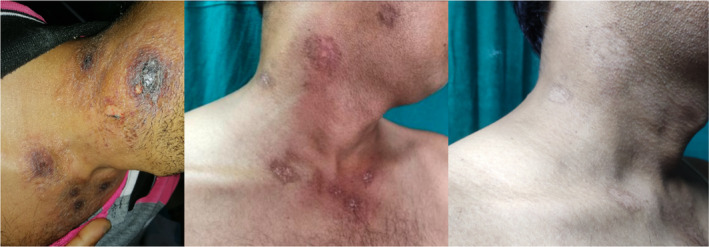
Multiple discrete target lesions over face, chest and neck during presentation, 3 weeks and 6 weeks after the initiation of treatment respectively.

**FIGURE 2:**
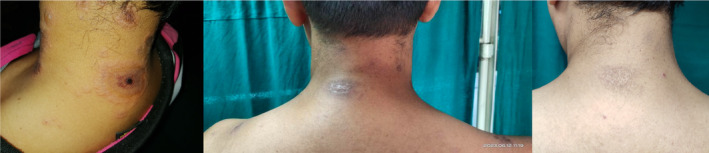
Target lesions over back of neck during presentation, 3 weeks and 6 weeks after the initiation of treatment respectively.

**FIGURE 3:**
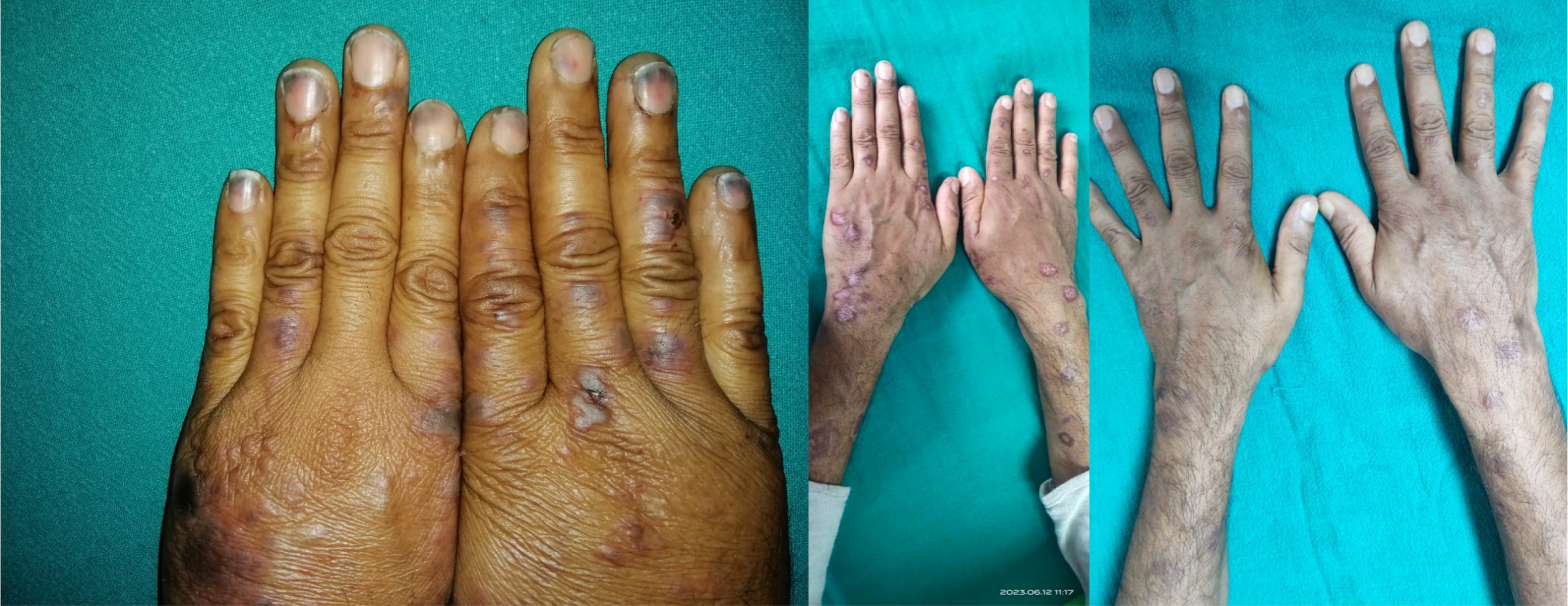
Multiple papules and target lesions of varying shapes over dorsum of hands and forearms during presentation, 3 weeks and 6 weeks after the initiation of treatment respectively.

Following the treatment, the skin lesions gradually resolved. The skin lesions during presentation, over a period of 3 weeks and 6 weeks, are shown in Figures 1, 2, 3.

## 5 CASE DISCUSSION

SLE is a chronic, recurrent, potentially fatal multisystem autoimmune and inflammatory connective tissue disorder whose diagnosis can be difficult due to the broad range of clinical manifestations and the lack of pathognomic features or specific laboratory tests.^7^, ^8^, ^9^ SLE can be fatal due to its potential to cause premature death, primarily because of active disease, organ failure (e.g., kidneys), infection, or cardiovascular disease resulting from accelerated atherosclerosis.^10^ Before puberty, the female‐to‐male ratio of SLE occurrence is 3:1; after puberty, the ratio increases to 9:1. SLE is generally classified into chronic cutaneous LE (CCLE), subacute cutaneous LE (SCLE), and acute cutaneous LE (ACLE).^7^, ^8^

The kidneys, brain, lungs, heart, skin, and joints are the major organs affected by SLE, with commonly presenting symptoms including fatigue, fever, arthralgias, myalgias, weight loss, rash, oral ulcers, thrombocytopenia, and leucopenia. The mainstay of laboratory testing for the diagnosis of SLE is the assessment of ANA. While a positive ANA test result is useful in diagnosis, it is not specific for SLE. In contrast, anti‐ds DNA is relatively specific for SLE.^9^ Up to 30% of patients will present with cutaneous symptoms, including butterfly‐shaped facial rash, red macules, papules, plaques, alopecia, and mucosal ulcers.^5^

The differential diagnoses for multiple system involvements include primary vasculitis such as Polyarteritis Nodosa (PAN), Wegner's granulomatosis (WG), or secondary vasculitis like systemic‐onset juvenile idiopathic arthritis (SOJIA), or other connective tissue diseases like SLE or dermatomyositis (DM).^7^ So, there is likely chance of misdiagnosis in the case of multi‐systemic involvements of case. In the case of the patient, the likely diagnosis of SLE was made based on the clinical presentations, including fever, oral ulcers, and a positive anti‐ds DNA.

EM, instead, is an acute, immune‐mediated mucocutaneous condition characterized by the presence of multiple symmetric, typical, or atypical target lesions with or without crusting at the center of the lesion and concentric color variation mainly on extremities (hands, feet, and the extensor aspects of limbs), with or without itching and prodromal symptoms.^8^ Classical or true EM is precipitated by trigger factors such as infective agents like herpes simplex virus, mycoplasma pneumoniae, drugs like anticonvulsants, antibiotics, and non‐steroid anti‐inflammatory drugs, any underlying malignancy, or connective tissue disorders, and is not associated with any specific serological abnormalities commonly seen in autoimmune disease or with chilblain.^6^, ^7^, ^8^ In the case of the patient, there was no identifiable precipitating cause of erythema multiforme that does not favor the diagnosis of true EM. Cases of EM associated with LE lesions where an EM trigger factor is missing are considered a diagnostic criterion for RS.^11^ Rowell syndrome was originally described in 1963 by Rowell et al., who identified four females with discoid LE, EM‐like lesions, and the presence of one of the following serology: speckled ANA, anti‐Ro/La antibody, or rheumatoid factor (RF).^1^ RS is characterized by the combination of EM, LE, and typical serological abnormalities.^12^

RS is an uncommon presentation of lupus erythematosus with erythema multiforme‐like lesions associated with specific serological changes, including positive rheumatoid factor (RF), speckled ANA, positive rheumatoid factor, or anti‐La antibodies in the serum.^8^, ^12^, ^13^ The speckled pattern of ANA is the most consistent diagnostic feature of Rowell's syndrome. Anti‐La antibodies and rheumatoid factor seem to be less consistent features.^6^ In our case, the patient had a positive RF and a positive ANA with a speckled pattern.

**FIGURE 4:**
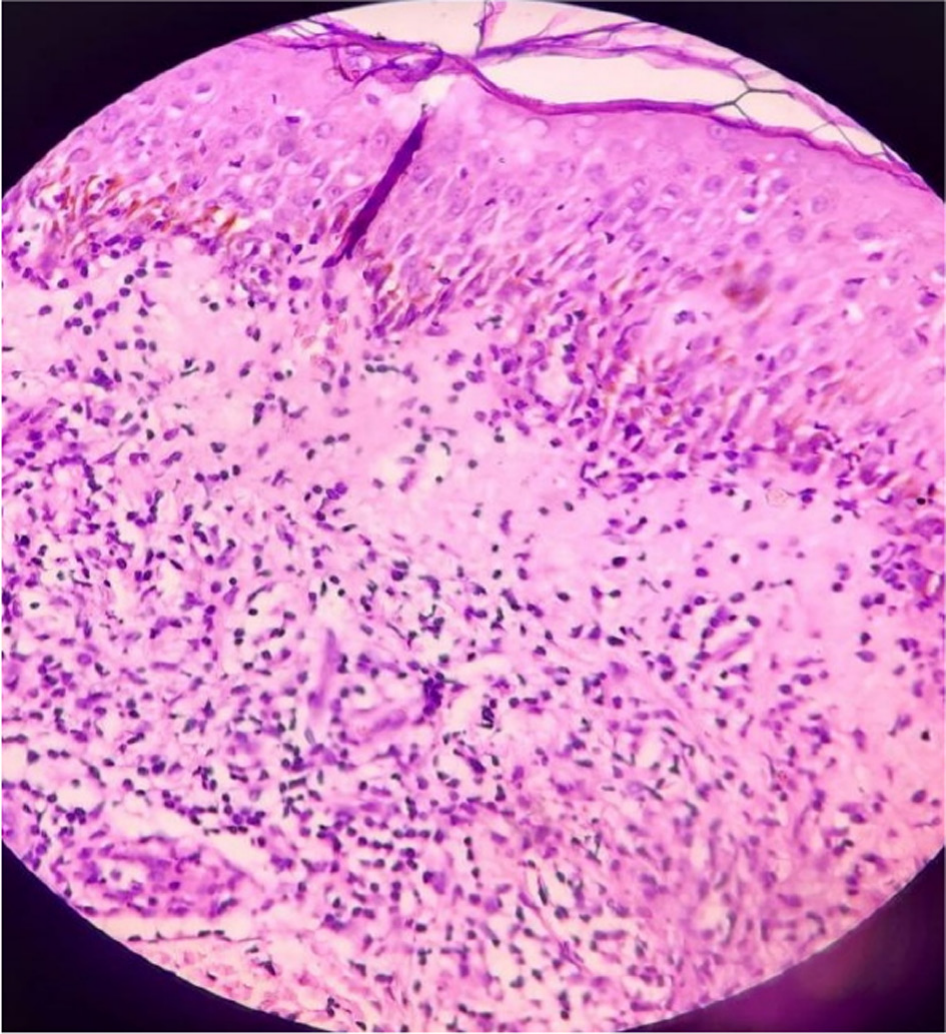
Skin biopsy section revealing areas of ulceration, follicular plugging, mixed inflammatory cells exocytosis, edematous and hemorrhagic dermis with thrombi formation suggestive of inflammatory dermatoses.

There is a question as to whether the EM‐like lesions of RS represent a subset of SCLE since vesicobullous lesions that resemble EM may occur rarely in SCLE. However, the vesicobullous lesions of SCLE do not result in clinical necrosis or scarring, and the histopathological features do not include necrosis of keratinocytes.^12^ Skin biopsy of the patient reveals areas of ulceration, follicular plugging, mixed inflammatory cells exocytosis, edematous, and hemorrhagic dermis with thrombi formation suggestive of inflammatory dermatoses as depicted in Figure 4.

In patients with SCLE, a positive ANA is seen in 75%; the pattern is usually homogenous. Positive anti‐Ro is found in 60% and a positive rheumatoid factor in 30%–40%. Immunofluorescence of lesional skin reveals linear IgA, IgM, and C3 at the dermo‐epidermal junction in 60% of patients. The immunofluorescence from an EM‐like lesion in our patient was negative.^12^

Before considering a diagnosis of RS, it is important to rule out common triggering agents and other differentials of EM. In this case, no precipitating factor for EM was identified. Also, a diagnosis of RS is based on the presence of major and minor criteria, as seen in Table 2.^1^, ^5^, ^6^, ^11^, ^14^ All three major and at least one minor criteria are required to confirm RS.^6^

**TABLE 2:** Criteria for RS diagnosis.^1^, ^5^, ^6^, ^10^, ^13^

Major criteria: Must meet all	Lupus Erythematosus (LE): systemic LE, discoid LE or subacute cutaneous LE Erythema multiforme‐like lesions with or without mucosal involvement Speckled pattern of antinuclear antibody
Minor criteria: Need at least 1	Positive rheumatoid factor Anti‐Ro antibody or Anti‐La antibody Chilblains

In our patient, as all the major criteria, along with one minor criterion, that is, positive RF, were present, we consider our case to be a classic RS. RS is a rare but distinct entity in rheumatology, and SLE presenting initially as EM‐like lesions is quite uncommon.^2^ Our case was an example of a similar situation where SLE initially presented as an EM‐like lesion. The pathological evaluation of the skin lesion might reveal EM and/or lupus erythematosus‐like manifestations.^3^ Similarly, the major pathological feature of our case includes both an EM and a LE lesion. Overall, in addition to being diagnosed with SLE and EM, our case fully meets the diagnostic criteria for RS.

There is no any standard treatment for RS.^1^ The therapeutic regimen used for RS and the prognosis are similar to those of SLE or DLE that occurs alone. However, the diagnosis becomes challenging when DLE coexists with SLE.^3^ The majority of the reported cases showed a satisfactory response to corticosteroids with azathioprine, antimalarial drugs such as chloroquine or hydroxychloroquine, dapsone, or cyclosporine.^1^, ^11^, ^13^ Studies have shown that Belimumab, a human monoclonal antibody directed against the B lymphocyte stimulator, is often considered effective in recalcitrant cases of SLE, although data regarding its use in cutaneous SLE is limited.^1^ The response to treatment can be assessed using the levels of certain autoantibodies (anti‐dsDNA, anti‐Nucl, anti‐His, and anti‐C1q), among which anti‐dsDNA performs the best when assessing disease activity, as suggested by the researchers Shang et al.^3^ Our patient was subsequently managed with hydroxychloroquine (200 mg once daily), prednisolone (1 mg/kg/day), steroid ointment, a proton pump inhibitor, and sunscreen cream after the diagnosis. Following the treatment, the skin lesions gradually resolve, as shown in Figures 1, 2, 3.

Case‐based review, including clinical and immunological characteristics of this case, and other similar cases of RS is shown in Table 3.

**TABLE 3:** Case‐based review showing clinical and immunological characteristics of this case and other patients with RS.

References	This case	Imtiaz et al., 2021 (1)	Li et al., 2023 (2)	Child et al., 1999 (3)	Chandra et al., 2020 (4)	Almansouri & Alzharani, 2020 (5)	Arevalo et al., 2020 (6)	Roy et al., 2013 (7)	Shadid et al., 2007 (8)	Gallo et al., 2020 (9)
Patient's age (in years)	41	66	41	29	18	30	20	10	87	17
Gender	M	F	F	F	F	F	M	M	F	M
Type	SLE	DLE	SLE	SCLE	DLE	SLE	SCLE	SLE	SLE	SLE
EM	+	+	+	+	+	+	+	+	+	+
MI	Yes	No	No	No	Yes	Yes	No	Yes	No	No
Perniosis	−	−	+	+	−	−	−	−	−	−
ANA	+, speckled pattern	+, homogenous pattern, 1:360 titer	+, speckled, 1:320	+, speckled	+, speckled	+	+	+, speckled pattern	+, homogenous pattern, 1:200 titer	+
Anti‐Ro	−	−	−	+	+	+	+	+	+	+
Anti‐La	−	−	−	+	−	+	+	+	+	Not mentioned
Anti‐ds DNA	+	+	+	−	Not mentioned	+	−	+	Not mentioned	+
RF	+	Not mentioned	Not mentioned	+	+	−	+	+	_	Not mentioned
Treatment	Hydroxychloroquine, prednisolone, steroid ointment	Hydroxychloroquine 200 mg, 0.05% halobetasol propionate cream, oral prednisolone, mycophenolate mofetil, belimumab	Methylprednisolone (1 mg/kg), prednisolone (40 mg/kg), hydroxychloroquine (400 mg/day)	Oral prednisolone, hydroxychloroquine (400 mg/day)	Oral prednisolone, hydroxychloroquine (200 mg/day)	Prednisolone 40 mg, azathioprine 150 mg, hydroxychloroquine, betamethasone, fusidic acid	Oral prednisolone	Methylprednisolone (30 mg/kg/day), oral prednisolone (2 mg/kg/day)	Prednisolone (20 mg/day), hydroxychloroquine (200 mg/day)	Prednisolone (1 m mg/kg), hydroxychloroquine 200 mg twice daily

Abbreviations: −, Negative; +, Positive; ANA, Antin Nuclear Antibody; DLE, Discoid Lupus Erythematosus; EM, Erythema Multiforme; F, Female; M, Male; MI, Mucosal Involvement; RF, Rheumatoid Factor; SCLE, Subacute Cutaneous Lupus Erythematosus; SLE, Systemic Lupus Erythematosus.

Our case is unique as the patient presented to us with features of EM, and on investigation, we found underlying systemic lupus erythematosus, thus SLE presenting for the first time as EM. Very rarely, SLE may initially present with recurrent episodes of EM‐like lesions. A high index of suspicion is needed for diagnosing RS when there is no evidence of a precipitating factor, and SLE should be considered in all patients presenting initially with EM‐like lesions. Early diagnosis and prompt treatment of SLE are required to prevent irreversible complications.

## 6 CONCLUSION

Our case emphasizes the importance of considering RS in all patients presenting initially as EM‐like lesions in the absence of triggering factors. Not being able to do so results in misdiagnosis of the disease, which will result in a delay in treatment and eventually irreversible organ damage and death. Certainly, clinicians need to be aware of the cutaneous signs associated with systemic diseases like SLE to facilitate early diagnosis and timely treatment before potentially life‐threatening complications develop. Hence, thorough and comprehensive research needs to be undertaken to establish the existence of RS as a separate entity.

## AUTHOR CONTRIBUTIONS

**Madhur Bhattarai:** Writing – original draft. **Niraj Kumar Sharma:** Writing – original draft. **Shreeram Paudel:** Writing – original draft. **Sujata Bhandari:** Writing – original draft. **Amrit Bhusal:** Writing – review and editing. **Kiran Dhonju:** Writing – review and editing. **Sandip Kuikel:** Writing – review and editing. **Shivendra Kumar Jha:** Writing – review and editing. **Egesh Aryal:** Writing – review and editing. **Deepak Subedi:** Writing – review and editing.

## FUNDING INFORMATION

There are no any sources of funding available for writing the manuscript and for the decision to submit the manuscript for publication.

## CONFLICT OF INTEREST STATEMENT

The authors have no conflict of interest to declare.

## CONSENT

Written informed consent was obtained from the patient to publish this report in accordance with the journal's patient consent policy.

## Data Availability

All the required information is available in the manuscript itself.

## References

[1] Imtiaz R , Hornback C , Eslam M , Crowson AN , Levin J . Targetoid lesions in a patient with systemic lupus erythematosus. Dermatol Online J. 2021;27(2):1‐7. 33818986

[2] Chandra A , Saha SK , Ray AK , Karmakar P . Rowell's syndrome: a rare but distinct entity in rheumatology. BMJ Case Rep. 2020;13(9):1‐4. 10.1136/bcr-2020-235173 PMC7513572 32967943

[3] Li Y , Cheng S , Ying C , Li L , Wang G , Chen X . Targetoid‐like lesions and chilblain‐like erythema manifested on hands and feet: a case of Rowell syndrome from China. Immunity, Inflamm Dis. 2023;11(8):1‐7. 10.1002/iid3.979 PMC10461425 37647424

[4] Almansouri AY , Alzahrani ZA . A case of rhupus with rowell syndrome. Open Access Rheumatol. 2020;12:91‐96. 32607016 10.2147/OARRR.S255790 PMC7293421

[5] Shadid NH , Thissen CACB , van Marion AMW , Poblete‐Gutiérrez P , Frank J . Lupus erythematosus associated with erythema multiforme: Rowell's syndrome. Int J Dermatol. 2007;46(SUPPL. 3):30‐32. 10.1111/j.1365-4632.2007.03508.x 17973886

[6] Zeitouni NC , Funaro D , Cloutier RA , Gagne E . Redefining Rowell's Syndrome. Br J Dermatol. 2000;142:343‐346. 10730772 10.1046/j.1365-2133.2000.03306.x

[7] Roy M , Ghosh JB , Bala AK , Chatterjee S . Rowell's syndrome: presenting features of systemic lupus erythematosus. Rheumatol Int. 2013;33(4):1075‐1077. 21152923 10.1007/s00296-010-1623-y

[8] An I , Harman M , Ibiloglu I . Topical Ciclopirox Olamine 1%: revisiting a unique antifungal. Indian Dermatol Online J. 2017;10(4):481‐485. 10.4103/idoj.IDOJ_29_19 PMC6615394 31334080

[9] Society for Maternal‐Fetal Medicine (SMFM) , Silver R , Craigo S , et al. Society for maternal‐fetal medicine consult series #64: systemic lupus erythematosus in pregnancy. Am J Obstet Gynecol. 2023;228(3):B41‐B60. 36084704 10.1016/j.ajog.2022.09.001

[10] Boedecker‐Lips SC , Weinmann‐Menke J . Systemic lupus erythematosus: what is new? Nephrol Ther. 2021;16(5):319‐330.

[11] Gallo L , Megna M , Festa B , et al. Rowell syndrome: a diagnostic challenge. J Clin Aesthet Dermatol. 2020;13(4):40‐42. PMC7605390 33144910

[12] Child FJ , Kapur N , Creamer D , Black AK . Rowell's Syndrome. Clin Exp Dermatol. 1999;24:74‐77. 10233657 10.1046/j.1365-2230.1999.00422.x

[13] Shahid S , Khan M , Qadar LT , Akmal M , Jamal A . The first case of Rowell syndrome with lupus nephritis and lobar pneumonia in a male Child reported in Pakistan. Cureus. 2019;11(5):7‐11. 10.7759/cureus.4604 PMC6609278 31309027

[14] Arevalo AB , Nassar R , Krishan S , Lakshmanan P , Salgado M , Chokshi P . Lupus never fails to deceive US: a case of Rowell's syndrome. Case Rep Rheumatol. 2020;2020:1‐4. 10.1155/2020/8884230 PMC7530503 33029442

